# In Vivo Regulation of Active Matrix Metalloproteinase-8 (aMMP-8) in Periodontitis: From Transcriptomics to Real-Time Online Diagnostics and Treatment Monitoring

**DOI:** 10.3390/diagnostics14101011

**Published:** 2024-05-15

**Authors:** Nur Rahman Ahmad Seno Aji, Tülay Yucel-Lindberg, Ismo T. Räisänen, Heidi Kuula, Mikko T. Nieminen, Maelíosa T. C. Mc Crudden, Dyah Listyarifah, Anna Lundmark, Fionnuala T. Lundy, Shipra Gupta, Timo Sorsa

**Affiliations:** 1 Department of Oral and Maxillofacial Diseases, Head and Neck Center, University of Helsinki and Helsinki University Hospital, 00290 Helsinki, Finland; 2 Department of Periodontics, Faculty of Dentistry, Universitas Gadjah Mada, Jalan Denta No. 1 Sekip Utara, 10 Sleman, Yogyakarta 55281, Indonesia; 3 Division of Pediatric Dentistry, Department of Dental Medicine, Karolinska Institutet, 171 77 Huddinge, Sweden; 4 Department of Otorhinolaryngology—Head and Neck Surgery, Helsinki University Hospital and University of Helsinki, 00290 Helsinki, Finland; 5 Wellcome-Wolfson Institute for Experimental Medicine, School of Medicine Dentistry and Biomedical Science, Queen’s University Belfast, Belfast BT9 7BL, UK; 6 Department of Dental Biomedical Sciences, Faculty of Dentistry, Universitas Gadjah Mada, Jl. Denta Sekip Utara No 1, Yogyakarta 55281, Indonesia; 7 Oral Health Sciences Centre, Post Graduate Institute of Medical Education & Research, Chandigarh 160012, India; 8 Division of Oral Diseases, Department of Dental Medicine, Karolinska Institutet, 171 77 Stockholm, Sweden

**Keywords:** active matrix metalloproteinase-8, aMMP-8, *Td*-dentilisin, periodontitis, transcriptomic, proteomic

## Abstract

Background: This study investigated in vivo regulation and levels of active matrix metalloproteinase-8 (aMMP-8), a major collagenolytic protease, in periodontitis. Methods: Twenty-seven adults with chronic periodontitis (CP) and 30 periodontally healthy controls (HC) were enrolled in immunohistochemistry and transcriptomics analytics in order to assess *Treponema denticola* (Td) dentilisin and MMP-8 immunoexpression, mRNA expression of MMP-8 and its regulators (IL-1β, MMP-2, MMP-7, TIMP-1). Furthermore, the periodontal anti-infective treatment effect was monitored by four different MMP-8 assays (aMMP-8-IFMA, aMMP-8-Oralyzer, MMP-8-activity [RFU/minute], and total MMP-8 by ELISA) among 12 CP (compared to 25 HC). Results: Immunohistochemistry revealed significantly more *Td*-dentilisin and MMP-8 immunoreactivities in CP vs. HC. Transcriptomics revealed significantly elevated IL-1β and MMP-7 RNA expressions, and MMP-2 RNA was slightly reduced. No significant differences were recorded in the relatively low or barely detectable levels of MMP-8 mRNAs. Periodontal treatment significantly decreased all MMP-8 assay levels accompanied by the assessed clinical indices (periodontal probing depths, bleeding-on-probing, and visual plaque levels). However, active but not total MMP-8 levels persisted higher in CP than in periodontally healthy controls. Conclusion: In periodontal health, there are low aMMP-8 levels. The presence of *Td*-dentilisin in CP gingivae is associated with elevated aMMP-8 levels, potentially contributing to a higher risk of active periodontal tissue collagenolysis and progression of periodontitis. This can be detected by aMMP-8-specific assays and online/real-time aMMP-8 chair-side testing.

## 1. Introduction

Periodontitis is a host-mediated, chronic inflammatory disease induced by dysbiotic bacterial biofilms and is characterized by progressive collagenolytic destruction and loss of the periodontal attachment and alveolar bone [1]. Within the bacterial biofilm, *Treponema denticola* (*Td*), an obligate anaerobe, is among the most well-characterized and frequently isolated spirochaetes associated with periodontitis [2]. One of its key virulence factors responsible for its high invasiveness is its cell surface-bound chymotrypsin-like proteinase (CTLP), also known as dentilisin [2]. *Td*-dentilisin can modulate host immunity and facilitate apoptosis in various cell types [2]. *Td*-dentilisin also degrades multiple host extracellular matrix and basement membrane (BM) proteins, hydrolyses non-matrix bioactive peptides and mediators, enhances *Td* penetration into the epithelium, activates pro-matrix metalloproteinases, and promotes its integration into biofilm communities [2]. Virulence factors of *Td* can trigger inflammatory and adaptive immune responses and increase the release and activation of MMP-8 [2,3,4]. This is mediated by the binding of pathogen-associated molecular patterns to pattern recognition receptors (toll-like receptors) of host inflammatory and resident cells [3]. The inflammatory cells (mainly neutrophils), resident fibroblasts, and epithelial cells in the periodontal tissues release proinflammatory mediators (interleukin-1β, tumor necrosis factor-alpha, prostaglandin E2, RANKL, etc.) and proteolytic enzymes, including MMPs, which can initiate the periodontal tissue destruction [3,5,6].

MMPs regulate the cell-matrix composition and hydrolyze the components of the ECM and BM, which are also potentially degraded to a lesser extent by microbial proteases [2,3,4]. MMPs also modify immune responses [4,7,8]. MMPs’ activities are mainly regulated by endogenous tissue inhibitors of matrix metalloproteinases (TIMPs), and the MMP/TIMP ratio frequently determines the extent of ECM protein degradation and tissue remodeling [3,4,7,8]. An imbalance in the MMPs/TIMP ratio is considered to tilt the balance toward pathological tissue destruction in periodontitis [3,4,7,8].

MMP-8 is the major collagenolytic protease present in both gingival crevicular fluid (GCF) and gingival tissue and is implicated in the inflammatory and immunological cascades in periodontitis [4,9,10,11,12,13,14]. MMP-8 can additionally process various non-matrix bioactive proteins such as cytokines, complement components, and insulin receptors [4,7]. The active form of MMP-8 (aMMP-8) is elevated in a diseased mouth rinse, gingival crevicular fluid, and peri-implant sulcular fluid samples and is potentially useful to diagnose, predict the stage and grade periodontitis/periimplantitis. Furthermore, it can act as a biomarker to differentiate periodontitis from gingivitis and a healthy state [9,14,15,16]. 

MMP-8 gene expression is regulated primarily at the transcriptional level during neutrophil development and maturation in the bone marrow [17,18,19,20,21], and the de novo up-regulation of its and other MMP’s mRNAs, in response to growth factors and cytokines in periodontitis and arthritis, has often been demonstrated [8,19,22]. Overexpression of MMPs necessitates the tight regulation of the collagenolytic and tissue-destructive MMP genes and proteins in periodontitis [4,8]. 

Recently, the activation of the host proMMP-8 by *Td*-dentilisin in patients with periodontitis and periimpantitis was reported [2,20]. Therefore, we hypothesized that increased *Td*-dentilisin could eventually invade and up-regulate aMMP-8 levels in periodontitis-affected tissues and oral fluids. 

In this study, we aimed to (i) detect *Td*-dentilisin and MMP-8 immunoexpression levels in gingival tissue samples of patients with periodontitis compared with periodontally healthy gingivae to (ii) assess the MMP-8, MMP-2, MMP-7, TIMP-1, and IL-1β mRNA expressions in the diseased vs. healthy gingiva. In addition, (iii) determine aMMP-8 and total MMP-8 levels before and after non-antibiotic anti-infective scaling and root planing treatment in chronic periodontitis (CP) in relation to healthy controls (HC), using independent aMMP-8 immuno—and catalytic activity assays.

## 2. Materials and Methods

### 2.1. Patients and Tissue Samples

The gingival tissue specimens were collected from stage III/IV grade B/C periodontitis (CP) patients (*n* = 27) and from periodontally healthy control (HC) patients (*n* = 30). The clinical dental examination and gingival tissue sample collection were approved by the Regional Ethics Board in Stockholm (number 2008/1935-31/3) and the local ethical committee of the Helsinki University Hospital, Finland (106§/26.06.2019; dnro HUS/1271/2019) and Regionala etikprövingsnämnden i Stockholm, (EPN) (2016-08-24/2016/1:8 and 2016-1-24; Dnr 2016/1410-31/1) in accordance with the Helsinki Declaration. All participants provided signed informed consent before enrolling in this study. The periodontitis tissue specimens were obtained from patients with generalized stage III/IV adult CP as diagnosed by a clinical assessment of pocket depths, loss of attachment, bone loss, and bleeding on probing [10]. The patients had radiographic alveolar bone loss in 30%-50% of teeth, loss of attachments between 5 and 7 mm, and elevated aMMP-8 (22–38 ng/mL) levels [10]. The patients had not received any antimicrobial or MMP-8 inhibitory low-dose doxycycline, bisphosphonate, chlorhexidine medication [3,4,7,8,19], or professional periodontal treatment of the sampling area prior to the participation of this study. Gingivitis or initial/early developing stage I periodontitis was defined clinically as the occurrence of redness, swelling of the gingiva, bleeding on probing, and aMMP-8 test positivity [1,9,10,15,16,23]. Gingivitis samples for this study were obtained from patients with gingival index < 2 and probing depth ≤ 3 mm without supporting soft and bone tissue destruction and recovered during gingivectomy in the case of gingival enlargement in the incisor, canine, or premolar sites [9,10,15,16,23].

Periodontitis-affected gingival tissue samples were collected during periodontal flap surgery and of gingivitis during gingivectomy. Healthy control tissue specimens from clinically non-inflamed gingiva were taken during the odontectomy of a fully embedded third molar. Although there were no clinically apparent signs of inflammation or pericoronitis, we cannot fully exclude the possibility that some control tissues might have been histologically slightly inflamed, as shown by the presence of some inflammatory cell infiltrates in the lamina propria [24]. The tissue sections were evaluated by an oral pathologist. The periodontitis tissue samples contained the oral, sulcular, and junctional epithelium and lamina propria beneath the epithelium, while healthy control tissues only contained oral epithelium and the lamina propria (since it was impossible to have the sulcular epithelium in this healthy tissue). Thus, we used sulcular epithelium and its lamina propria for periodontitis, while for the healthy tissues, we used its oral epithelium and lamina propria. Additionally, 5 periodontitis-affected gingival tissue samples contained dental plaque biofilm adjacent to the tissue, and the immunoexpression of Td-dentilisin in this plaque was evaluated in addition to the gingival tissue. The biopsy samples were carefully selected according to specific criteria, followed by an evaluation based on histological findings.

### 2.2. Immunohistochemical Analysis

From the collected gingival tissue samples, periodontitis-affected (*n* = 9) and healthy (*n* = 10) tissue specimens were formaldehyde-fixed, processed, and paraffin-embedded for immunohistochemistry. Histological staining was performed on the paraffin-embedded gingival tissue biopsies. Serial sections (4 µm) were deparaffinized using xylene and rehydrated through ethanol series. Sections of each biopsy were histologically stained with hematoxylin and eosin in order to assess the orientation of the tissue structures. Immunoexpression of Td-dentilisin and MMP-8 in the gingival tissue sections was determined by immunohistochemical staining with Td-dentilisin (1:1500 rabbit polyclonal IgG, as described by Al-Samadi et al. [24] and 6 µg/mL MMP-8 rabbit polyclonal antibody [25], respectively. Sections for MMP-8 and Td-dentilisin immunostainings were subject to antigen retrieval using the same following procedure. After deparaffinization, the antigens were retrieved in a citrate buffer using microwaves (MicroMED T⁄T Mega Histoprocessing Labstation; Milestone Srl, Sorisole, Italy). Endogenous peroxidase activity was inhibited with 3% H_2_O_2_ in PBS for 15 min. To inhibit non-specific staining, slides were incubated for 1 h at room temperature in normal goat serum from the Vectastain^®^ kit 1:10 in 0.1% BSA-PBS. Slides were then incubated with primary Ab (anti-MMP-8 and Td-dentilisin [1:3000] antibodies) overnight at +4 °C. Biotinylated anti-rabbit IgG from Vectastain^®^ kit (Vector Laboratories, Burlingame, CA, USA) was used as a secondary antibody (1:200 dilutions in 0.1% BSA-PBS. Slides were then incubated in avidin–biotin–peroxidase complexes. The color was developed in 0.006% H_2_O_2_ containing 0.023% 3,3′-diaminobenzidine tetrahy-drochloride (DAB) chromogen for 10 min. Slides were washed in PBS three times, with 5 min between each step [24]. The staining of Td-dentilisin was graded as 0 (negative, [9,10,15,16,23]), 0,5 (very low, [+/−]), 1 (low, [+]), 2 (moderate, [++]), and 3 (strong, [+++]). MMP-8 was scored as 0 (negative, [9,10,15,16,23]), 1 (low, [+]), 2 (moderate, [++]), and 3 (strong, [+++]).

### 2.3. RNA Sequencing and Transcriptomics of Gingival Tissue Biopsies

The gingival tissue samples collected from patients with adult chronic stage III/IV grade B/C CP (*n* = 18) and the healthy controls (HC, *n* = 20) were processed for transcriptomic analysis (TRNSCRMS). For periodontitis, the classification and inclusion criteria were radiographic bone resorption, clinical attachment level 5–7 mm, tooth sites with probing depth (PPD) ≥6 mm, and enhanced bleeding on probing representing stage III/IV-grade B/C-periodontitis [1,10,11]. For healthy control subjects, the inclusion criteria were no sign of periodontal disease, no gingival/periodontal inflammation, probing depth ≤ 3.0 mm, clinical attachment level ≤ 3.0 mm, and no bleeding on probing [1,10]. This study was approved by the Regional Ethics Board in Stockholm (number 2008/1935-31/3). 

Total RNA was isolated using the Qiagen RNeasy kit (VWR, Stockholm, Sweden). The quality of RNA was assessed using the RNA 6000 NanoLabChip kit of the Bioanalyzer system from Agilent Technologies (Santa Clara, CA, USA). The RNA libraries were prepared and sequenced using the Illumina stranded TruSeq protocol. This involved capturing polyA-RNA with polyT-coated magnetic beads, RNA fragmentation, reverse transcription, second strand synthesis with dUTP incorporation, ligation of sequencing adapters, and PCR amplification of adapter-ligated fragments, following Illumina’s provided instructions. The sequence alignment and analysis were performed, as previously described [26].

**Figure 1:**
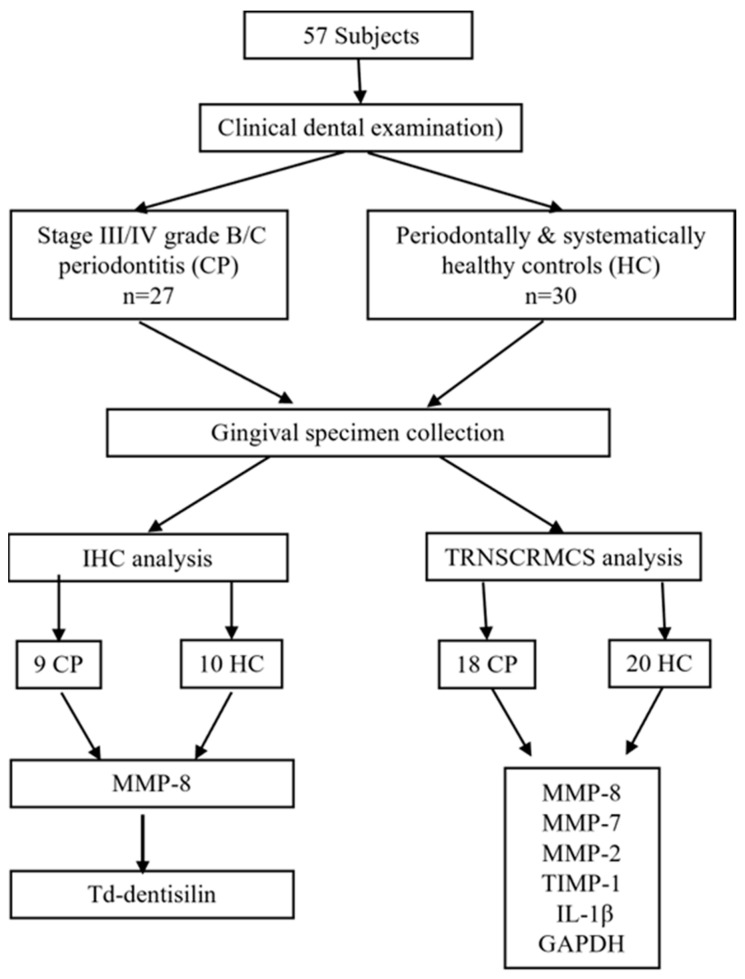
Flow chart of immunohistochemical (IHC) and transcriptomic (TRNSCRMS) analyses.

The RNA-seq data for the selected genes, including MMP-8, MMP-7, MMP-2, TIMP-1, and IL-1β, were further analyzed for differential expressions in CP and HC samples. Additionally, the housekeeping genes glyceraldehyde 3-phosphate-dehydrogenase (GAPDH) was included in the analysis [26]. A flow chart of transcriptomic and immunohistochemical analysis is provided in Figure 1.

### 2.4. Periodontal Anti-Infective Scaling and Root Planing Treatment

Comprehensive periodontal examination, non-antibiotic anti-infective scaling, and root planing periodontal treatment were carried out by a single periodontist. After aMMP-8 POCT test and clinical full mouth recordings at baseline (t0), anti-infective full-mouth scaling and root planing treatment procedures were performed along with oral hygiene instructions for 12 CP patients stage III/IV-grade B/C, a separate set of CP patients [1,9,10]. At 5 (t1) and 10 (t2) weeks after the aMMP-8 POCT testing and the full-mouth clinical examination, anti-infective periodontal treatment was carried out again. The 23–25-year-old systemically and periodontally healthy dental students, who were enrolled as healthy controls (HC, had an aMMP-8 POCT test and full-mouth clinical examination.

1. Chairside PoC and quantitative aMMP-8 analyses;
2. aMMP-8 levels were measured online and in real time quantitatively by a rapid PoC chairside aMMP-8 kits (Periosafe^®^, Dentognostics GmbH, Solingen, Germany) and a quantitative reader (Oralyzer^®^, Dentognostics GmbH, Solingen, Germany) from the collected mouth rinse samples from both the periodontitis patient group (*n* = 12) and the healthy control group of 25 systemically and periodontally healthy dental students. Any remaining oral mouth rinse fluid was transferred to Eppendorf tubes and stored at −70 °C for further laboratory analysis [9,10];
3. Measurement of the aMMP-8 Levels by Immunofluorometric Assay (IFMA)

The aMMP-8 levels from mouth rinse samples were also determined by a time-resolved immunofluorescence assay (IFMA), as described previously [9]. Briefly, aMMP-8-specific monoclonal antibodies 8708 and 8706 (Actim Oy, Espoo, Finland) were used in the analysis as capture and tracer antibodies, respectively. In this protocol, the diluted samples were allowed to incubate for 1 h with the Europium-labeled tracer antibody. The fluorescence was measured using an EnVision 2015 multimode reader (PerkinElmer, Turku, Finland) [10].

### 2.5. MMP-8 Activity Assay Using Relative Fluorescence Units/Min (RFU)

An MMP-8 activity assay was adapted from the protocol of McCrudden et al. (2017) with slight modifications [27]. The wells of Greiner^®^ 96-well black high binding plates (Merck, Darmstadt, Germany) were coated with 100 μL/well MMP-8 capture antibody (Merck Millipore, Watford, UK), at a concentration of 2 µg/mL in 0.05 M carbonate buffer, pH 9.6. The plate was covered and incubated at 4 °C overnight. The contents of the wells were discarded the following day, and plates were washed three times with phosphate-buffered saline (PBS, pH 7.4) containing 0.05% (*v*/*v*) Tween-20 (PBST). A blocking step was then carried out with 200 µL of PBST containing 1% (*w*/*v*) bovine serum albumin (BSA) at room temperature for 1 h. Wells were washed three times with PBST and incubated at room temperature for 2 h with 100 µL/well GCF samples or recombinant MMP-8 standard (Bio-Techne, Abingdon, UK). Recombinant MMP-8, supplied in its proform, was activated (as directed by manufacturers) by pre-treatment with 1 mM 4-aminophenylmercuric acetate (APMA) for 1 h at 37 °C prior to use in the MMP-8 activity assay. All GCF samples (prepared at a dilution factor of 1:4), as well as the APMA-activated MMP-8 standards (3.125–100 ng/mL), were diluted in AnaSpec MMP assay buffer (AnaSpec, Fremont, CA, USA) prior to analysis in the MMP-8 activity assay. Duplicate preparations of all samples and standards were carried out in the assay. Following this incubation step, plates were washed three times with PBST. To each well, 45 μL AnaSpec MMP assay buffer was added, followed by 45 μL of 10 µM AnaSpec 520 MMP fluorescence resonance energy transfer (FRET) substrate SB-XIV (AnaSpec, Fremont, CA, USA). Prior to use, the FRET substrate was reconstituted to 1 mM in dimethyl sulfoxide (DMSO) and diluted to 100 μM in MMP Assay Buffer (AnaSpec, Fremont, CA, USA). The substrate was then stored in aliquots at −20 °C. Following the addition of the MMP Assay Buffer and FRET substrate to the wells of the plate, fluorescence measurements were recorded immediately at excitation and emission wavelengths of 485 nm and 525 nm, respectively. Measurements were recorded over a 70 min period, at 5 min intervals, on a microtitre plate reader (Genios, Tecan, Reading, UK) using Magellan software Version 7.2 (Tecan, Reading, UK), and the results were displayed as relative fluorescence units (RFU) per minute (RFU/min) [27].

### 2.6. Statistical Analysis

MMP-8 and *Td*-dentilisin immunoexpressions (IHC) and transcriptomic (TRNSCRMS) analysis of MMP-8, MMP-7, MMP-2, TIMP-1, IL-1β parameters were calculated by an independent samples *t*-test was performed to assess the significance of differences between CP and HC group in all recorded parameters. The periodontal anti-infective treatment effect, i.e., differences in the levels of the four different MMP-8 assays (aMMP-8 IFMA, aMMP-8 Oralyzer, MMP-8 activity [RFU/minute], and total MMP-8 by ELISA) and the clinical parameters between t0, t1, and t2 were tested with Friedman’s test (asymptotic, 2-sided), followed by pairwise post hoc comparisons by Dunn–Bonferroni test. A two-tailed *p*-value < 0.05 was considered statistically significant. Data management and statistical analysis were performed by utilizing spreadsheet software (Microsoft Excel for Mac 16.78) and the SPSS version 29.0 (IBM SPSS Statistics for Windows, IBM Corp., Armonk, NY, USA).

## 3. Results

### Ex Vivo Immunoexpression of Td-Dentilisin and MMP-8 in Human Periodontitis-Affected vs. Healthy Gingival Tissues

**Figure 2:**
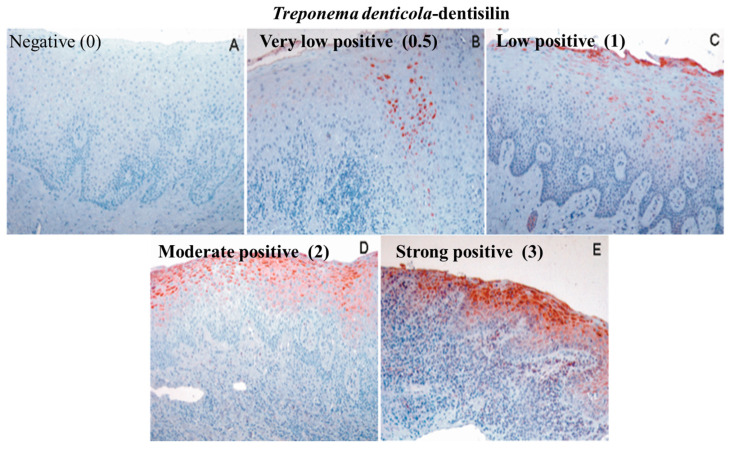
Immunoexpression of *Td*-dentilisin. Gingival tissue specimens were graded and scored as (**A**) negative (0), (**B**) very low-positive (0.5), (**C**) low-positive (1), (**D**) moderate-positive (2), and (**E**) strong-positive (3). The gingival tissue specimens (**A**,**B**) represent periodontitis classification stage I /grade A, specimens (**B**,**C**) stages I–II grade B, and specimens (**D**) stages III–IV/grade C, respectively. The chromogen (red) was 3-amino-9-ethylcarbazole (AEC), and the counterstain was hematoxylin. *Td*-dentilisin was detected in all red-stained regions on each tissue segment. Magnification 200×.

**Figure 3:**
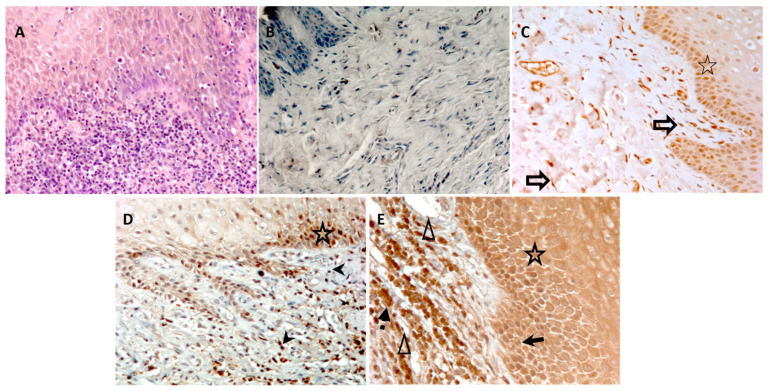
Immunoexpression of MMP-8. (**A**) Hematoxyline eosin staining of the periodontitis gingival tissue. (**B**) Immunohistochemical staining of the gingival tissues is scored as negative (0). (**C**) Low-positive (scored 1). (**D**) Moderate-positive (scored 2). (**E**) Strong-positive (scored 3). The gingival tissue specimen (**C**) represents periodontitis classification stages O–I/grade A; specimen (**D**) represents stages II–III grade B; specimen (**E**) represents stages III–IV grade C. The cells show MMP-8 expression, including epithelial cells (star), neutrophils (black arrow), lymphocytes (arrowhead), macrophage (black arrow with dash), endothelial cells (blank arrowhead/triangle), and fibroblasts (blank arrow) of periodontitis gingival tissues. DAB is used as a chromogen (brown) and hematoxyline as a counterstain. All brown-stained areas on each tissue section indicate specific detection of MMP-8. Magnification 200×.

Gingival tissue specimens were stained with antibodies to *Td*-dentilisin and MMP-8 to visualize immunoexpressions in CP gingiva compared with HC gingiva. The immunostainings of the *Td*-dentilisin and MMP-8 are shown in Figure 2 and Figure 3, respectively. *Td*-dentilisin could be detected most clearly and intracellularly intensively in the gingival epithelium relative to lamina propria. *Td*-dentilisin immunoexpressions increased according to the severity of periodontitis (stages and grades), indicating the evident invasion route of *Td* and its dentilisin–protease from the superficial dental plaque biofilm into deeper periodontal tissues in vivo. The immunoexpressions of *Td*-dentilisin were significantly stronger in CP-affected tissues than in HC-gingivae (*p* < 0.05) (Figure 2, Table 1). The majority of CP-affected tissues expressed low to moderate *Td*-dentilisin immunopositivity along with the increase in clinical disease severity, while HC-gingivae were negative or had hardly detectable *Td*-dentilisin immunoexpression (score 0–0.5) (Figure 3, Table 1).

In CP, MMP-8 immunoexpression was significantly higher in lamina propria compared to epithelium (*p* < 0.05). Lamina propria expression of MMP-8 was higher in CP compared with HC-gingivae (*p* < 0.05). Immunoexpression of MMP-8 in CP-gingivae increased according to the increase in periodontal disease severity (stages and grades). There are no detectable differences in MMP-8 expressions observed between epithelium and lamina propria in HC-gingivae. 

The normalized counts for the genes MMP-8, MMP-7, MMP-2, TIMP-1, and IL-1β in CP and HC are demonstrated in Table 1. In CP-gingivae (stages III/IV, grades B/C), the translations of IL-1β and MMP-7 were significantly increased. The elevation of TIMP-1 transcription was noticed without reaching statistical significance (Table 1). MMP-2 translation was reduced in CP-gingivae vs. HC-gingivae without statistical significance. MMP-8 translation was low and similar, barely detectable in either CP-gingivae or HC-gingivae (Table 1). The expression of the housekeeping gene GAPDH was similar in CP- and HC-gingivae (Table 1).

**Figure 4:**
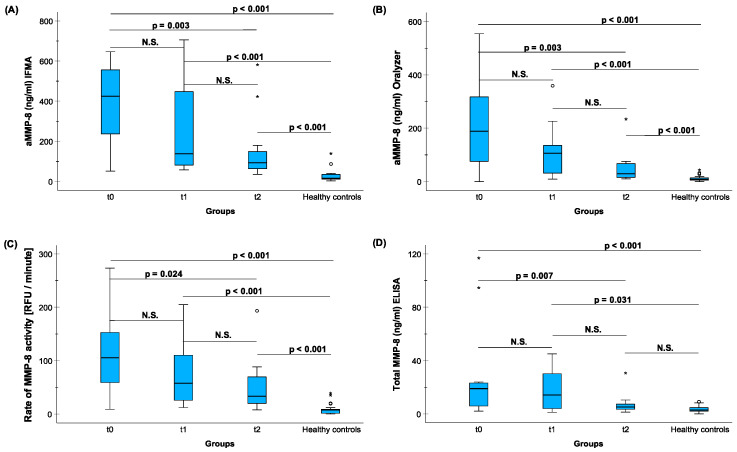
The treatment effects of anti-infective treatment in 12 CP-patients to the aMMP-8 and total MMP-8 levels assessed by four different a/tMMP-8 assays. (**A**) Active matrix metalloproteinase-8 (aMMP-8) (ng/mL) IFMA; (**B**) aMMP-8 (ng/mL) Oralyzer; (**C**) MMP-8 activity assay (RFU per minute); and (**D**) total MMP-8 (ng/mL) ELISA vs. levels in HC. Patients were examined based on baseline level at t0, 1st recall visit t1 (5 weeks), and 2nd recall visit t2 (10 weeks). The differences in a/t MMP-8 assay levels between t0, t1, and t2 were tested with Friedman’s test (asymptotic, 2-sided) (**A**) *p* = 0.005, (**B**) *p* = 0.005, (**C**) *p* = 0.017, and (**D**) *p* = 0.009; and pairwise post hoc comparisons by Dunn–Bonferroni test are marked in the plots. The differences between 25 HCs (healthy controls) and 12 CP-patients in t0, t1, and t2 in the four different MMP-8 assay levels calculated by Bonferroni-corrected Kruskal–Wallis test are marked in the plots. Asterisk (*) and circle (o) represent outliers of more than 3 times the interquartile range and between 1.5 and 3 times the interquartile range, respectively.

**Figure 5:**
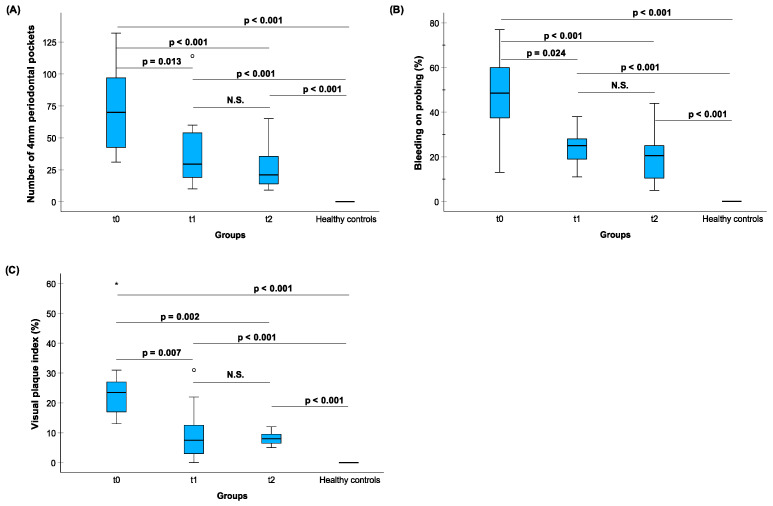
The treatment effects of anti-infective treatment in 12 CP patients to the recorded clinical periodontal parameters. (**A**) The number of at least 4mm periodontal pockets; (**B**) bleeding on probing (%), and (**C**) visual plaque index (%) in relation to HCs. Patients were examined at base level t0, 1st recall visit t1 (5 weeks), and 2nd recall visit t2 (10 weeks). The differences in the clinical parameters between t0, t1, and t2 were tested with Friedman’s test (asymptotic, 2-sided) (**A**) *p* < 0.001, (**B**) *p* < 0.001, and (**C**) *p* < 0.001; and pairwise post hoc comparisons by Dunn–Bonferroni test are marked in the plots. The differences between 25 HCs (healthy controls) and 12 CP-patients at t0, t1, and t2 in the recorded clinical indices calculated by Bonferroni-corrected Kruskal–Wallis test are marked in the plots. Asterisk (*) and circle (o) represent outliers of more than 3 times the interquartile range and between 1.5 and 3 times the interquartile range, respectively.

The treatment effects of anti-infective treatment (scaling and root planing) in 12 patients with stages III/IV grades B/C CP-patients were monitored by four different MMP-8 assays (aMMP-8 IFMA, aMMP-8 Oralyzer, rate of MMP-8 activity [RFU per minute] and total MMP-8 ELISA) (Figure 4). Clinical periodontal parameters were also measured (Figure 5). Furthermore, these two figures present the successful treatment’s effect assessed by both clinical parameters and by MMP-8 assays’ levels of CP-patients vs. HCs. aMMP-8 assays (IFMA, Oralyzer, aMMP-8 activity/RFU assay) more precisely than total MMP-8 assay demonstrated and reflected the clinically beneficial reducing effects of the anti-infective treatment. Furthermore, when comparing MMP-8 assay levels of 12 CP-patients to 25 HCs revealed that only total MMP-8 levels could reach healthy control levels, while aMMP-8 IFMA, aMMP-8 Oralyzer, and aMMP-8 activity (RFU per minute) assays all decrease significantly but did not reach those observed in healthy subjects (Figure 4 and Figure 5).

## 4. Discussion

In the present study, we addressed the regulation of MMP-8 expression in vivo in chronic adult periodontitis gingiva vs. healthy gingiva by immunohistochemical and transcriptomic (TRNSCRMS) analysis. In addition, we compared various aMMP-8 and total MMP-8 assays as adjunctive diagnostic tools to monitor their levels in mouth rinse in periodontal treatment vs. systemically and periodontally healthy controls. MMP-8, also known as neutrophil collagenase (collagenase-2), has been regarded to be solely released by human neutrophilic leukocytes [19,20,21], but the protease and its RNA have also been identified in the non-neutrophil-lineage cells, such as human articular chondrocytes, synovial and gingival fibroblasts, endothelial cells, odontoblasts and T-cell line as well as malignant cells [8,19,22]. Differing from MMP-1 and -2, which are constitutively de novo-transcribed and expressed by various non-malignant and malignant mesenchymal-type cells, MMP-8, after maturation in the bone marrow in latent proform, is prepacked and stored in subcellular specific granules in mature circulating neutrophils (PMNs) [4,7,8,19,20,21]. MMP-8 regulation at the sites of inflammation is thus regarded to occur mainly through the selective PMN degranulation and activation of the released latent proMMP-8 to active MMP-8 (aMMP-8) [18,19,20,21]. In periodontitis, periodontal pathobionts and their proteolytic virulence factors can effectively induce the selective PMN-degranulation and related proteolytic activation of latent proMMP-8 to aMMP-8 in vitro [19,20,21]. 

Immunohistochemical results of the present study revealed that the presence of *Td*-dentilisin in CP-gingival tissues was increasingly associated with the increase in MMP-8 immunoexpression along with an increase in clinical stage and grade disease severity of periodontitis. Our present results, thus, further support and extend the concept that the *Td*-dentilisin can eventually invade from the dysbiotic dental plaque biofilm into the diseased periodontitis-affected gingival tissue and up-regulate the degranulation of neutrophils and related activation of the released latent proMMP-8 to aMMP-8 [2,20]. The increased MMP-8 immunoexpression was found not only in epithelium but also in lamina propria. This showed that the inflammation occurred both in epithelium and lamina propria. The inflammatory immune response is triggered by the interaction of resident cells with the bacterial biofilm attached to the tooth surface, including *Td*. The epithelium, especially junctional epithelium, is the first periodontal structure to face the *Td* invasion. Dentilisin produced by *Td* facilitates this spirochaeta to invade and penetrate the deeper epithelium layer [28], stimulating the gingival epithelial cells and the underlying cell in lamina propria to trigger the initial inflammatory responses. The inflammatory response will activate host cells to produce MMP-8 as one of the inflammatory mediators. The MMP-8 secretory cells, being mainly infiltrating neutrophils and also other reported potential non-PMN-lineage cellular sources of MMP-8 in the diseased human inflamed gingiva, including epithelial cells, resident fibroblasts, endothelial cells, and mononuclear inflammatory cells, as shown in this study. 

In degranulating mature circulating neutrophils, the de novo expression of MMP-8 cannot be induced as the gene expression is carried out during the neutrophil’s maturation in the bone marrow and the latent/inactive MMP-8 is stored in its granules [17,18,19,21], but in non-PMN-lineage cells, such fibroblasts, endothelial cells, chondrocytes and epithelial cells, de novo expression of MMP-8 and its mRNA is inducible at the sites of inflammations. Our present transcriptomic data of MMP-8 mRNA and its potential regulators (IL-1β, MMP-7, and TIMP-1) mRNAs revealed that MMP-8 RNA was not transcriptionally up-regulated in CP-gingivae vs. HC-gingivae. On the other hand, it is known that the MMP-8′s up-regulator’s IL-1β mRNA [19,22] and activator MMP-7 mRNA [29] and potential endogenous inhibitor TIMP-1 mRNA can be de novo transcriptionally up-regulated in the diseased periodontitis gingivae [4,7,8,30,31]. Enhanced transcriptional and inductive IL-1β and MMP-7 expressions [4,7,8,30,31] in periodontitis-affected gingiva can eventually potentiate and complement the *Td*-dentilisin-mediated microbial-dependent activation of the degranulated latent proMMP-8 to aMMP-8 [20]. TIMP-1 mRNA up-regulation in the diseased gingiva eventually reflects the host’s endogenous defense, attempting to inhibit the elevated MMP-8 and -7 [31]. 

Previous in vitro studies have shown that IL-1β and other proinflammatory cytokines can up-regulate MMP-8 and its RNA [19,22]. The de novo transcriptomic in vitro expression of MMP-8 and its RNA in articular chondrocytes, gingival, and synovial fibroblast, as well as endothelial cells, has been demonstrated [19,22]. MMP-7 and its RNA can transcriptionally be up-regulated by proinflammatory mediators, including IL-1β and TNF-α previously detected in increasing amounts in diseased and inflamed tissues, including gingiva and synovium [30,31]. Nonetheless, previous in vivo studies have also revealed rather low or barely detectable de novo transcriptional expression of MMP-8 RNA in the diseased periodontitis-affected gingiva and peri-apical periodontitis-affected lesions [31,32,33]. Our present ex vivo MMP-8 mRNA transcriptomic findings support and further extend those previous in vivo [32,33,34] rather than the in vitro studies [17,19,22] revealing rather low de novo transcriptional expression of MMP-8 RNA in the periodontitis-affected gingiva vs. healthy gingiva [32,33,34]. Our present data support the conjuncture that cytokine (IL-1β) induced neutrophil extravasation and selective degranulation together with periodontopathogenic-dependent (*Td*-dentilisin) activation of the released proMMP-8 to aMMP-8 contributes to periodontal tissue destruction [19,20].

The potential benefit and usefulness of utilizing aMMP-8 POCT as the biomarker in the new periodontitis staging and grading categorization have been demonstrated by Sorsa et al. [9], Keskin et al. [10], Deng et al. [15,16], and Sahni [35], as well independently confirmed by Deng et al. [31,32]. The implementation of aMMP-8 as the selected biomarker in the new periodontitis classification was successfully confirmed and further extended in the current study [7,9,10,15,16]. Furthermore, we showed that aMMP-8 levels in HC mouth rinses were significantly lower (i.e., below 20 ng/mL) than aMMP-8 levels in CP patients. These systemically and periodontally healthy adults (HCs) were 23–25-year-old dental students with very good oral health habits who had never experienced periodontal disease [10], and their low aMMP-8 levels indicated no/low risk of collagenolytic disease activity in the near future. Noteworthy, in HCs, the low MMP-8 levels do not represent active MMP-8 but instead represent mainly total latent proMMP-8. In that regard, Ganghar et al. [36], Overall et al. [37], Lee et al. [38], Mancini et al. [39], and Romanelli et al. [40] have shown that in oral fluid of periodontally healthy patients, the MMP-8 is latent proMMP-8 rather than aMMP-8. 

In the present study, it was observed that only total MMP-8 decreased back to a healthy control level due to the treatment effect. However, aMMP-8 levels, despite being reduced significantly due to the non-antibiotic anti-infective scaling and root planing treatment affecting and reflecting clinical indices, were not reduced back to healthy control levels (Figure 4). It is possible that when periodontitis develops to stages III/IV grade B/C periodontitis, it is difficult, or almost impossible thereafter, for active collagenolytic activity to reduce back to healthy levels again. Doxycycline, an aMMP-8 inhibitor, can aid in reducing MMP-8 activation but not completely [41]. Furthermore, Romanelli et al. [40], Mancini et al. [39], Gellibolian [42], and Overall et al. [37] have demonstrated by using different and independent collagenase activity assay and immunoassays that the major type of MMP-8 in progressive periodontitis lesions is aMMP-8 and not latent total proMMP-8. MMP-8 is not activated and fragmented in gingivitis, but it is activated and fragmented in periodontitis [40]. In this regard, many studies using total MMP-8 as the oral fluid periodontitis and periimplantitis biomarker have failed. Noteworthy, aMMP-8 is collagenolytic and proteolytic, whereas total latent proMMP-8 is neither collagenolytic nor proteolytic [19,21]. Therefore, it is an aMMP-8 and not a total MMP-8 [43,44,45], which reflects clinically active and progressive periodontitis in oral fluids [9,10,38,39,40]. And hence, moving ahead, aMMP-8 should not be regarded as synonymous with total MMP-8 in periodontitis diagnosis [35].

These findings strongly suggest that low aMMP-8 levels (<20 ng/mL), as detected by aMMP-8 POCT, may be regarded as a biomarker of periodontal health [9,10], as determined by independent aMMP-8 catalytic activity assay [27], as well as by aMMP-8 IFMA immunoassay utilizing the same aMMP-8 selective monoclonal antibody [10], as in the aMMP-8 POC test [9,10,35]. All three independent aMMP-8 assays were found to correlate with each other well, and all also reflect clinical indices of periodontal disease before, during, and after successful anti-infective periodontal treatment. 

## 5. Conclusions

Consistent with similar independent tests for aMMP-8, such as the catalytic aMMP-8-RFU activity assay [27] and aMMP-8 IFMA [10], the use of aMMP-8 POCT in chair-side applications lasting only 5 min has been shown to be convenient [9,15,16]. It serves as a reliable method for real-time quantitative diagnostics and ongoing monitoring during periodontal treatment with scaling and root planing, supplementing its effectiveness. Analysis of aMMP-8 levels following successful scaling and root planing treatment indicates a significant reduction, approaching levels observed in healthy individuals. This contrasts with total MMP-8, which lacks precision as a biomarker for periodontitis. Levels of aMMP-8 can be influenced by microbial proteases, such as those released by *Td*, which trigger specific release of neutrophils in the gingiva affected by chronic periodontitis (CP), as well as by the direct action of *Td*-dentilisin, activating MMP-8 to aMMP-8. This mechanism, rather than de novo expression of neutrophil MMP-8 in the gingiva, is a key contributor to tissue damage in CP. The use of aMMP-8 POCT is, thus, advantageous as a supplementary diagnostic, point-of-care, treatment-monitoring, and preventive tool in adult chronic periodontitis.

## Figures and Tables

**Table 1:** Immunoexpression of *Td*-dentilisin, MMP-8, and RNA expressions of MMP-8 and its regulators MMP-7, MMP-2, TIMP-1, and IL-1β in CP-stages III/IV grades B/C gingivae vs. HC gingivae. In IHC, *n* = 10 for HC, and *n* = 9 for CP, and in RNA sequencing/transcriptomics, *n* = 20 for HC, and *n* = 18 for CP.

Parameters	Healthy (HC)	Periodontitis (CP)	Significance (*p*-Value)
*Immunohistochemical staining of gingival tissue*			
*Td*-dentilisin	1.00 ± 0.21	2.44 ± 0.06	*, *p* = 0.0038
MMP-8	1.00 ± 0.20	4.33 ± 0.41	*, *p* = 0.0001
*RNA sequencing/transcriptomics*			
MMP-8	0.35 ± 0.62	0.46 ± 0.84	-
MMP-7	1.78 ± 4.17	49.92 ± 106.16	*, *p* = 0.0280
MMP-2	191.27 ± 138.23	147.05 ± 138.66	-
TIMP-1	47.28 ± 16.62	66.01 ± 49.47	-
IL-1β	14.73 ± 16.78	71.47 ± 61.32	*, *p* = 0.0001
GAPDH	2491.42 ± 630.29	2596.36 ± 840.13	

* = significantly different, *p* < 0.05, *t*-test. Abbreviations: MMP-8 = matrix metalloproteinase 8; MMP-7 = matrix metalloproteinase 7; MMP-2 = matrix metalloproteinase 2; TIMP-1 = tissue inhibitor matrix metalloproteinase 1; IL-1β = interleukin 1 beta; GAPDH = glyceraldehyde 3-phosphate-dehydrogenase; HC = healthy control; CP = chronic periodontitis.

## Data Availability

Data supporting reported that the results can be obtained from the authors on request.
